# Factors associated with oral health behaviors of older diabetic patients in Shanghai, China: a structural equation modeling analysis based on the COM-B model

**DOI:** 10.3389/fpubh.2026.1823813

**Published:** 2026-06-17

**Authors:** Shuran Fan, Yanhong Gu

**Affiliations:** 1 Shanghai Fifth People’s Hospital, Fudan University, Shanghai, China; 2 Center of Community-Based Health Research, Fudan University, Shanghai, China; 3 School of Nursing, Fudan University, Shanghai, China

**Keywords:** association pathways, older diabetic patients, oral health behaviors, structural equation modeling, the COM-B model

## Abstract

**Aims:**

The study aims to analyze the association pathways of factors related to oral health behaviors among older diabetic patients in Shanghai, China, using the COM-B model.

**Methods:**

A convenience sample of 358 older diabetic patients was enrolled between July 2025 and December 2025 from one tertiary hospital and a community health service center in Shanghai. Information was collected via a general information questionnaire, the Older Oral Health Knowledge, Belief and Behavior Questionnaire, the 14-item Health Literacy in Dentistry Scale, the Perceived Social Support Scale, the Geriatric Self-Efficacy for Oral Health Scale, and the 5-item Geriatric Depression Scale. Descriptive statistics, univariate analysis, correlation analysis, and path analysis were adopted to analyze the relevant factors and pathways of oral health behaviors.

**Results:**

The constructed structural equation model showed satisfactory goodness of fit (χ^2^/df = 2.173, NFI = 0.987, TLI = 0.976, CFI = 0.991, RMSEA = 0.07, and SRMR = 0.0474). Capability factors, including oral health literacy (*β* = 0.205) and oral health knowledge (*β* = 0.155), the opportunity factor of social support (*β* = 0.169), motivational factors covering oral health self-efficacy (*β* = 0.377), oral health belief (*β* = 0.225), and depression (*β* = −0.107), could all be directly associated with oral health behaviors. Oral health self-efficacy mediated the associations of oral health literacy (*β* = 0.199), oral health knowledge (*β* = 0.072), social support (*β* = 0.049), and depression (*β* = −0.044) with oral health behaviors. Oral health belief mediated the relationships of oral health literacy (*β* = 0.054) and oral health knowledge (*β* = 0.135) with oral health behaviors. Depression acted as a mediator between social support (*β* = −0.056) and oral health behaviors.

**Conclusion:**

Capability factors, opportunity factors, and motivational factors are directly or indirectly associated with the oral health behaviors among older diabetic patients. This suggests that clinicians regularly conduct diabetes-related oral health education and cleaning skills training, encourage relatives to provide supervision, improve access to oral healthcare, and provide psychological counseling to improve patients’ oral health behaviors in terms of capability, opportunity, and motivation.

## Introduction

The prevalence of diabetes among Chinese older adults aged ≥60 years is approximately 30% (1, 2). Due to the dual effects of aging and the disease, the prevalence of oral diseases in this population is as high as 90% (3, 4). Oral health is not only associated with the nutritional status and aging process of older diabetic patients but is also closely related to their blood glucose control and the occurrence of complications, making it an important part of their health management (3). Oral health behaviors are a series of individual activities aimed at maintaining oral health, including oral cleaning and oral disease management. As a crucial modifiable determinant, oral health behaviors play a significant role in preventing and controlling systemic risks related to oral diseases (5).

Identifying the factors related to oral health behaviors helps explain the interrelationships and provides evidence for developing oral health interventions for older diabetic patients. Prior studies have shown that individuals’ oral health self-efficacy (6), oral health literacy (6), oral health knowledge (7), oral health belief (7), social support (8), and depression (9) are correlated with oral health behaviors. A Thai study on older oral health behaviors indicated that their self-efficacy, oral health literacy, and social support were correlated with oral health behaviors (6). The findings by Zhang et al. (7) showed that oral health knowledge and belief among the older in pension institutions were both positively linked to their oral health behaviors. Jiang et al. (8) demonstrated that the greater social support of people, the better their oral health behaviors. Li (10) reported that oral health literacy and oral health self-efficacy are associated with oral health behaviors in older diabetic patients. Nevertheless, inconsistent findings exist regarding some associated factors. Turner et al. (11) reported that 58% of depressed individuals brushed their teeth less than once daily, markedly lower than the 12% in the control group. Conversely, Almohaimeed et al. (12) detected no significant impact of depression on oral hygiene practices or dental care utilization. Although existing studies have focused on oral health behaviors in older diabetic patients, they still have certain limitations. Most relevant studies only explore the independent association of single factors, such as oral health literacy and self-efficacy, on oral health behaviors. They fail to integrate the interrelationships among various factors, nor do they systematically explore the comprehensive association pathways of multidimensional factors based on a theoretical framework, which cannot effectively support the formulation and implementation of targeted oral health intervention measures.

The Capability, Opportunity, Motivation-Behavior (COM-B) model asserts that the occurrence of individual behavior requires three indispensable conditions: capability, opportunity, and motivation. All three are directly associated with individual behavior, and capability and opportunity are also indirectly correlated with behavior through motivation (13). It provides a theoretical foundation for analyzing the pathways of factors associated with individual behavior. Capability factors refer to the physical and psychological abilities that individuals possess to engage in a specific behavior; opportunity factors refer to all external factors that facilitate the occurrence of an individual’s behavior, including physical opportunities and social opportunities; motivation factors refer to the mental processes in the brain that motivate and guide behavior, including reflexive motivation and autonomous motivation.

**Figure 1:**
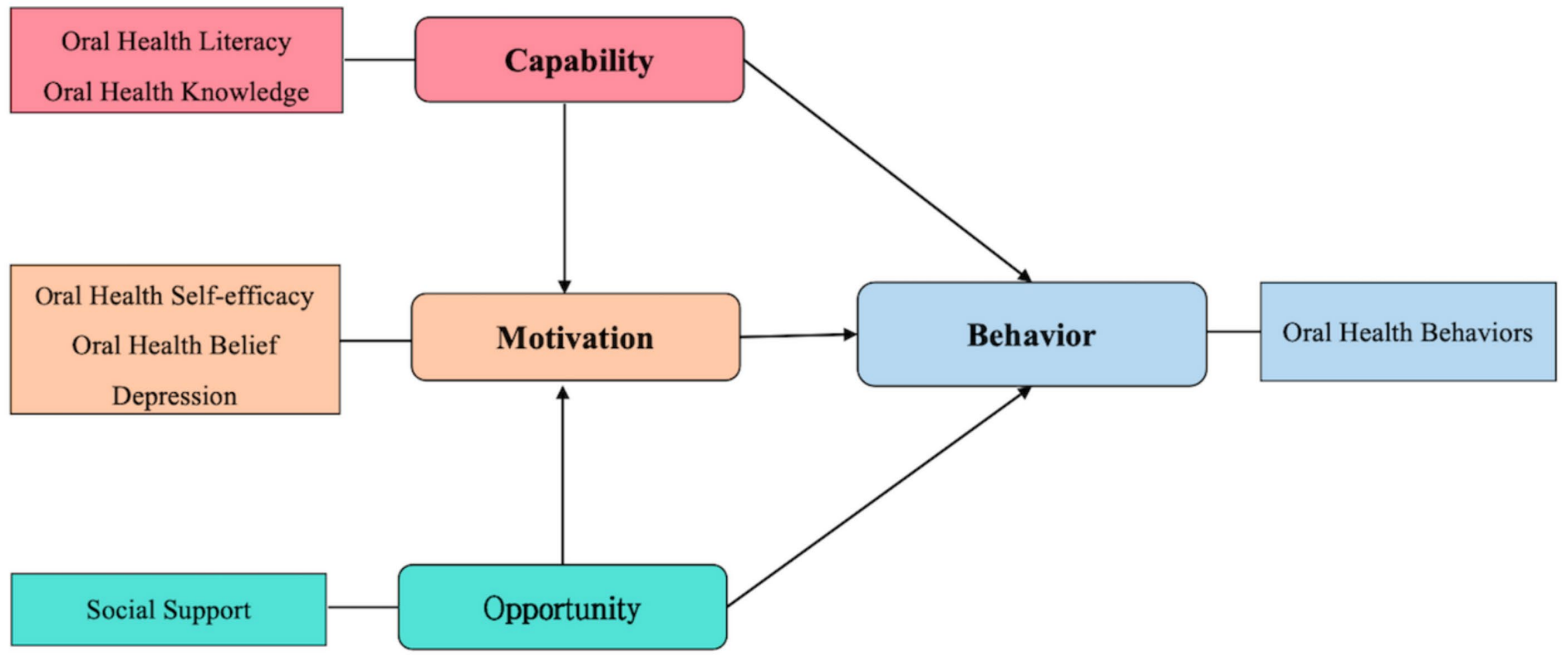
Conceptual model of factors related to oral health behaviors in older diabetic patients.

Therefore, we employed structural equation modeling to analyze relationships among variables (Figure 1), and proposed the following research hypotheses based on the COM-B model:

*Hypothesis 1*: Capability factors (oral health literacy and oral health knowledge), opportunity factors (social support), and motivation factors (oral health self-efficacy, oral health belief, and depression) are directly associated with oral health behaviors in older diabetic patients.

*Hypothesis 2*: Motivation factors (oral health belief, oral health self-efficacy, and depression) mediate the relationships between capability factors (oral health literacy, oral health knowledge) and oral health behaviors in older diabetic patients.

*Hypothesis 3*: Motivation factors (oral health belief, oral health self-efficacy, and depression) mediate the relationships between opportunity factors (social support) and oral health behaviors in older diabetic patients.

Focusing on older patients with diabetes mellitus, this study constructed a structural equation model based on the COM-B model to analyze the relationships and association pathways among the patients’ capabilities (oral health knowledge and oral health literacy), opportunities (social support), motivations (oral health belief, oral health self-efficacy, and depression), and oral health behaviors. This study aims to fill the gaps in previous research and provide references for interventions aimed at improving oral health behaviors and enhancing oral health among older diabetic patients.

## Methods

### Design and participants

From July 2025 to December 2025, we recruited older diabetic patients who received medical services at one tertiary hospital and a community health service center in Shanghai using convenience sampling. Inclusion criteria: (1) Satisfying the diagnostic requirements for type 2 diabetes: classic symptoms; accompanied by random blood glucose ≥ 11.1 mmol/L, or fasting blood glucose ≥ 7.0 mmol/L, or 2-h plasma glucose of oral glucose tolerance test (OGTT) ≥ 11.1 mmol/L, or HbA1c ≥ 6.5% (14); (2) Diagnostic course of disease ≥ 1 month; (3) Aged ≥ 60 years old (1); (4) Patients with normal verbal communication, hearing and vision, and ability to cooperate in completing the research survey; (5) Voluntarily taking part in this research and signing the informed consent. Exclusion criteria: (1) Patients in critical condition who are unable to cooperate; (2) Patients with oral malignant tumors or acute oral infections; (3) Patients with severe cognitive dysfunction who are unable to understand and complete the questionnaire. Sample size estimation: The sample size calculation formula was $\;n={\left[(Z_{\alpha /2}\times \sigma )/\delta \right]}^{2}$ , σ refers to the population standard deviation of oral health behaviors scores among older diabetic patients, and *δ* is the allowable error. We set α = 0.05 and Z_α/2_ = 1.96. In this study, σ = 10.86 was adopted from a previous study by Zhang et al. (7) on oral health behaviors among the older in Shanghai, and δ = 1.2. We computed a target sample size of 315. Allowing for a 10% non-response rate, the minimum sample size calculated in this study was 350 cases. And the requirement that the sample size for structural equation modeling should be no less than 200 cases, and for complex structural equation models, the ratio of sample size to free parameters should be no less than 10:1 (15). Our study included three exogenous observed variables (oral health literacy, oral health knowledge, and social support), three mediating observed variables (oral health self-efficacy, oral health belief, and depression), one endogenous latent variable of oral health behaviors, and two observed indicators (oral cleaning behaviors and oral condition management behaviors). Accordingly, the original hypothetical model contained a total of 25 free parameters, including 15 structural paths, 3 exogenous covariance terms, 6 residual variances, and 1 factor loading. The required sample size was no less than 250. Therefore, a sample size of at least 350 was required.

### Measurements

#### General information questionnaire

The questionnaire was developed independently by the researcher based on the research purpose and relevant literature review. It was then revised through collective discussion by the research team before being applied, with 15 items. General demographic data: Covering gender, age, marital status, educational level, smoking and drinking status, average monthly household income, occupation type, place of residence, and medical payment method. Disease-related information: Including disease duration, blood glucose control level, number of complications, family history of diabetes, and comorbid chronic conditions.

#### The older oral health knowledge, belief, behavior questionnaire

This questionnaire was developed by Ye et al. (16), focusing on three aspects of oral health in the older: knowledge, belief, and behavior, consisting of three sub-questionnaires. Knowledge dimension: Covers four sub-dimensions, including manifestations of oral problems, factors affecting oral health, oral hygiene practices, and oral hygiene products, with 19 items, and the overall score spanned 0 to 19. Belief dimension: Includes 4 sub-dimensions, namely oral health benefits, severity of oral health issues, lifestyle-related susceptibility to oral problems, and impediments to maintenance of oral health, with 17 items, and the overall score spanned 0 to 17. Behavior dimension: Comprises 2 sub-dimensions, including oral condition management behaviors and oral cleaning behaviors, with 11 items. The overall score spans 11 to 55 points, and a higher score is indicative of better oral health knowledge, belief and behaviors. In our study, the Cronbach’s *α* values of these three sub-dimensions of the scale are 0.827, 0.816, and 0.856, showing good reliability; the KMO value was 0.819, 0.874, 0.869, and Bartlett’s test of sphericity was significant (χ^2^ = 1042.965, 1322.641, 1609.937, *p* < 0.001), indicating that the data was suitable for exploratory factor analysis. The confirmatory factor analysis indicated good model fit (χ^2^/df = 1.152, 1.250, 2.653, NFI = 0.904, 0.902, 0.950, TLI = 0.902, 0.979, 0.942, CFI = 0.941, 0.978, 0.941, RMSEA = 0.038, 0.027, 0.069, and SRMR = 0.0391, 0.0472, 0.0428), suggesting they all had satisfactory construct validity. All dimensions had CR > 0.7 and AVE ≥ 0.5, indicating good composite reliability and convergent validity of the three sub-questionnaires. The square root of the AVE of each dimension was greater than the correlation coefficient between the dimension and other dimensions, suggesting good discriminant validity.

### 14-item health literacy in dentistry scale (HeLD-14)

Initially constructed by Jones et al. (17), the scale was localized for Chinese contexts by Yan et al. (18). It consists of seven dimensions with two items in each dimension, covering acceptance, understanding, support, medical treatment, financial burden, application, and communication. A 5-point Likert scale was adopted for assessment, and the overall score spanned 0 to 56 points. Higher scores reflect a greater oral health literacy. In our study, the Cronbach’s *α* coefficient is 0.908, showing good reliability; the KMO value was 0.869, and Bartlett’s test of sphericity was significant (χ^2^ = 4246.023, *p* < 0.001), indicating that the data was suitable for exploratory factor analysis. The confirmatory factor analysis indicated good model fit (χ^2^/df = 2.085, NFI = 0.973, TLI = 0.973, CFI = 0.986, RMSEA = 0.056, and SRMR = 0.0466) suggesting the scale had satisfactory construct validity. All seven dimensions had CR > 0.7 and AVE ≥ 0.5, indicating satisfactory composite reliability and convergent validity for each dimension of the scale. The square root of AVE for each dimension exceeded its correlation coefficients with other dimensions, indicating satisfactory discriminant validity.

### Perceived social support scale (PSSS)

The scale was constructed by Zimet et al. (19), which is utilized to measure subjective perception and experience of social support among individuals. This scale is composed of three dimensions, encompassing 12 items in total: four items assessing family support, four items evaluating friend support, and four items measuring other forms of support. Assessment was conducted using a 7-point Likert scale, and the overall score ranged between 12 and 84 points. Greater score represented stronger social support. In our study, it demonstrated a Cronbach’s *α* of 0.938, showing good reliability; the KMO value was 0.887, and Bartlett’s test of sphericity was significant (χ^2^ = 4761.438, *p* < 0.001), indicating that the data was suitable for exploratory factor analysis. The confirmatory factor analysis indicated good model fit (χ^2^/df = 2.694, NFI = 0.974, TLI = 0.977, CFI = 0.983, RMSEA = 0.069 and SRMR = 0.0262), suggesting the scale had satisfactory construct validity. All three dimensions had CR > 0.7 and AVE ≥ 0.5, indicating satisfactory composite reliability and convergent validity for each dimension of the scale. The square root of AVE for each dimension exceeded its correlation coefficients with other dimensions, indicating satisfactory discriminant validity.

### Geriatric self-efficacy for Oral health scale (GSEOH)

Initially constructed by Ohara et al. (20), it was localized for Chinese contexts by Xu et al. (21). This scale is composed of three dimensions with a total of 20 items: oral hygiene habit (8 items), oral functioning (9 items), and dental visit habit (3 items). A 4-point Likert scale was adopted for scoring, and the overall score spanned 20 to 80. Greater scores indicate higher oral health self-efficacy. In our study, it demonstrated a Cronbach’s *α* of 0.934, showing good reliability; the KMO value was 0.880, and Bartlett’s test of sphericity was significant (χ^2^ = 3736.469, *p* < 0.001), indicating that the data was suitable for exploratory factor analysis. The confirmatory factor analysis indicated good model fit (χ^2^/df = 2.786, NFI = 0.901, TLI = 0.906, CFI = 0.929, RMSEA = 0.071, and SRMR = 0.0766), suggesting the scale had satisfactory construct validity. All three dimensions presented CR > 0.7 and AVE ≥ 0.5, indicating satisfactory composite reliability and convergent validity for each dimension of the scale.

### 5-item geriatric depression scale (GDS-5)

This scale was simplified by Hoyl et al. (22) on the basis of the 15-item Geriatric Depression Scale (GDS-15) and is applied to assess depressive symptoms in the older. It consists of five items, with all responses scored as “Yes” or “No” using a dichotomous scoring system. Except for Item 1, which is reversely scored, all other items score 1 point for a “Yes” and 0 points for a “No.” Ranging from 0 to 5, the total scale score indicates depressive symptoms when ≥ 2, and a higher score denotes more serious depressive symptoms. In our study, it demonstrated a Cronbach’s *α* of 0.800, showing good reliability; the KMO value was 0.810, and Bartlett’s test of sphericity was significant (χ^2^ = 653.082, *p* < 0.001), indicating that the data was suitable for exploratory factor analysis. The confirmatory factor analysis indicated good model fit (χ^2^/df = 2.581, NFI = 0.980, TLI = 0.976, CFI = 0.988, RMSEA = 0.067,and SRMR = 0.0287), suggesting the scale had satisfactory construct validity. All dimensions had CR > 0.7 and AVE ≥ 0.489, indicating that each dimension of the scale possessed good composite validity. Although several AVEs were slightly below the threshold of 0.5, the convergent validity was still acceptable. The square root of the AVE of each dimension was greater than the correlation coefficient between that dimension and other dimensions, suggesting good discriminant validity of the scale.

### Data collection

After obtaining approval from the hospital’s relevant departments, trained researchers conducted the survey before data collection began. Considering that the majority of the subjects were older adults, face-to-face interviews were adopted for data collection. After obtaining the subjects’ consent, the investigators filled out the questionnaires on behalf of the subjects.

### Ethical considerations

The ethical principles of the Declaration of Helsinki were strictly followed in this study, with approval obtained from the Ethics Committee of the Fifth People’s Hospital of Shanghai (No. 2025291).

### Data analysis

Data were double-entered and cross-checked for accuracy. Using IBM SPSS Statistics 25.0, the Kolmogorov–Smirnov (K-S) normality test combined with the method of moments was used to analyze the distribution characteristics of the data (23). Data conformed to a normal distribution with K-S normality test *p* > 0.05, by the method of moments, data satisfied approximate normal distribution when absolute value of skewness < 2 and absolute value of kurtosis < 3. Descriptive statistics summarized patients’ characteristics normally distributed data or approximately normal distributed data were reported as mean and standard; adopting Cronbach’s *α* coefficient to test the internal consistency reliability of the research tools; KMO test and Bartlett’s sphericity test were adopted to judge whether the data was suitable for factor analysis; confirmatory factor analysis was used to test the construct validity, composite reliability, and discriminant validity of the tools. Independent-samples t-test or one-way ANOVA was used. Pearson correlation analysis was applied to investigate associations among variables. Multicollinearity among variables was assessed to examine potential collinearity issues. Combined with the results of correlation analysis, statistical analyses were performed via AMOS 26.0, and when building the SEM, the exogenous variables were permitted to correlate with each other to control for potential confounding associations. The criteria for evaluating model fit were as follows: the ratio of chi-square to degrees of freedom (χ^2^/df) < 3, root mean square error of approximation (RMSEA) < 0.08, Standardized Root Mean Square Residual (SRMR) < 0.08, comparative fit index (CFI) ≥ 0.90, Tucker-Lewis index (TLI) ≥ 0.90, and normed fit index (NFI) ≥ 0.90. Path association decomposition test was performed using the bias-corrected Bootstrap method with 5,000 iterative sampling times. The 95% confidence interval (CI) excluding zero served as the primary judgment criterion, and the *p* < 0.05 indicated significant path effects. The initial model was gradually modified and optimized based mainly on the maximum likelihood ratio, combined with modification indices (MI), standardized residuals, and theoretical rationality.

## Results

### Participant characteristics and univariate analysis

This study distributed 358 questionnaires in total, and 353 valid ones were retrieved, with an effective response rate of 98.60%. Among 353 participants, 274 aged 60–74 years, 79 aged ≥ 75 years; 178 female (50.4%), 175 male (49.6%). Univariate analysis revealed statistically significant differences in the scores of oral health behaviors across different subgroups of age, gender, marital status, educational level, per capita monthly household income, occupation, medical insurance type, disease duration, comorbidities, blood glucose control level, and comorbid chronic conditions (*p* < 0.05), see Table 1. Multiple post-hoc comparisons were performed on the factors with statistically significant differences, including education level, per capita monthly household income, disease course, number of complications, and blood glucose control level. The results showed that the total score of oral health behaviors presented a significant upward trend with the increase of educational level and per capita monthly household income. Patients with a disease course of more than 20 years had significantly lower scores than other disease course groups. The score of patients with 0–1 complications was significantly higher than that of those with 2–3 complications and > 3 complications. The well blood glucose control group had a significantly higher score than the moderate and poor blood glucose control groups (*p* < 0.05).

**Table 1:** Sample characteristics and univariate analysis of oral health behaviors in older diabetic patients (*N* = 353).

Variable	*N* (%)	Score (M ± SD)	t/F	*p*
Age				
60–74 years old	274 (77.6)	34.24 ± 9.90	2.062	0.041*
≥75 years old	79 (22.4)	31.71 ± 8.82		
Gender				
Male	175 (49.64)	34.72 ± 10.44	2.062	0.04*
Female	178 (50.4)	32.61 ± 8.81		
Marital status				
Currently married	308 (87.3)	34.12 ± 9.64	2.267	0.023*
Not currently married	45 (12.7)	30.60 ± 9.775		
Educational level				
Illiterate	28 (7.9)	25.39 ± 9.00	30.463	<0.001**
Primary school	52 (14.7)	29.06 ± 9.79		
Junior High School	111 (31.4)	30.59 ± 7.60		
Senior High School	105 (29.7)	37.49 ± 8.06		
University and above	57 (16.1)	40.93 ± 8.88		
Smoking status				
Yes	69 (19.5)	31.67 ± 7.41	−1.922	0.055
No	284 (80.5)	34.45 ± 10.24		
Drinking status				
Yes	46 (13)	34.09 ± 8.01	0.309	0.758
No	307 (87)	33.61 ± 9.71		
Monthly per capita income				
Below 5,000 yuan	131 (37.1)	23.63 ± 8.02	81.491	<0.001**
5,001–10,000 yuan	160 (45.3)	35.42 ± 8.02		
Above 10,000 yuan	62 (17.6)	42.50 ± 7.93		
Occupation type				
Mental work	133 (37.7)	38.17 ± 9.04	7.244	<0.001**
Physical work	220 (62.3)	30.96 ± 9.09		
Place of residence				
Rural area	9 (2.5)	29.00 ± 8.26	−1.711	0.144
Urban	344 (97.5)	33.80 ± 9.73		
Medical insurance				
Urban residents medical insurance	318 (90.1)	34.45 ± 9.58	5.320	<0.001**
Rural cooperative medical scheme	35 (9.9)	26.66 ± 8.06		
Duration of illness				
1–4 years	80 (22.7)	33.94 ± 10.99	4.921	0.002*
5–10 years	77 (21.8)	36.01 ± 10.00		
11–20 years	129 (36.5)	34.02 ± 9.10		
>20 years	67 (19)	30.00 ± 7.88		
Comorbidities				
0–1 type	125 (35.4)	37.54 ± 8.96	24.909	<0.001**
2–3 types	136 (38.5)	31.07 ± 8.98		
>3 types	92 (26.1)	28.48 ± 10.85		
Blood glucose control level				
Good	125 (35.4)	37.56 ± 9.12	17.09	<0.001**
Moderate	136 (38.5)	31.85 ± 9.69		
Poor	92 (26.1)	31.10 ± 8.92		
Family history of diabetes				
Yes	168 (47.6)	33.88 ± 9.46	0.382	0.704
No	185 (52.4)	33.49 ± 9.95		
Comorbid chronic conditions				
0–1 type	200 (56.7)	35.23 ± 10.04	3.485	<0.001**
≥2 types	153 (43.3)	31.65 ± 8.90		

*p < 0.05, **p < 0.001. Bold: Statistically significant.

### Scores and correlation analysis

According to the K-S normality test, the scores for the Oral Health Behavior Questionnaire were normally distributed (*p* > 0.05). Distribution characteristics of the Oral Health Knowledge Questionnaire, HeLD-14, GDS-5, PSSS, GSEOH, and Oral Health Belief Questionnaire were analyzed using the method of moments. Results indicated that all scale scores approximately followed a normal distribution (absolute skewness < 2, absolute kurtosis < 3). Therefore, all data were presented as mean ± standard deviation. The scores of the Oral Health Behavior Scale, HeLD-14, Oral Health Knowledge Scale, PSSS, GSEOH, Oral Health Belief Scale, and GDS-5 in older patients with diabetes mellitus were 33.67 ± 9.71, 36.95 ± 9.23, 12.53 ± 3.88, 52.27 ± 14.84, 50.62 ± 7.45, 12.29 ± 3.55, and 0.74 ± 1.33, respectively, (see Table 2). Pearson correlation analysis results showed that oral health behaviors were positively correlated with oral health literacy (*r* = 0.787), oral health knowledge (*r* = 0.727), social support (*r* = 0.713), oral health self-care efficacy (*r* = 0.815), and oral health belief (*r* = 0.717), and negatively correlated with depressive symptoms (*r* = −0.501). All correlations reached statistical significance at *p* < 0.001 (see Table 3).

**Table 2:** Scores of oral health behaviors, capability factors, opportunity factors and motivation factors in older diabetic patients (*N* = 353).

COM-B model	Items	Number of items	Score range	Total score (M ± SD)	Mean item score (M ± SD)
Behavior	Oral health behaviors	11	11–55	33.67 ± 9.71	3.06 ± 0.88
	Oral condition management behaviors	7	7 ~ 35	19.80 ± 6.74	2.83 ± 0.96
	Oral cleaning behaviors	4	4 ~ 20	13.88 ± 3.90	3.47 ± 0.97
Capability	Oral health literacy	14	0–56	36.95 ± 9.23	2.64 ± 0.66
	Oral health knowledge	19	0–19	12.53 ± 3.88	0.66 ± 0.20
Opportunity	Social support	12	12–84	52.27 ± 14.84	4.36 ± 1.24
Motivation	Oral health self-efficacy	20	20–80	50.62 ± 7.45	2.53 ± 0.37
	Oral health belief	17	0–17	12.29 ± 3.55	0.73 ± 0.21
	Depression	5	0–5	0.74 ± 1.33	0.15 ± 0.27

**Table 3:** Correlation between oral health behaviors and scores of capability, opportunity, and motivation factors in older diabetic patients (*N* = 353).

Variables	1	2	3	4	5	6	7
1. Oral health behaviors	1						
2. Oral health literacy	0.787**	1					
3. Oral health knowledge	0.727**	0.633**	1				
4. Social support	0.713**	0.695**	0.560**	1			
5. Oral health self-efficacy	0.815**	0.789**	0.644**	0.656**	1		
6. Oral health belief	0.717**	0.623**	0.755**	0.556**	0.586**	1	
7. Depression	−0.501**	−0.408**	−0.372**	−0.411**	−0.483**	−0.340**	1

**Indicates a statistically significant association at the 0.001 level (two-tailed).

### Multicollinearity diagnosis

The results of multicollinearity diagnosis showed that the Variance Inflation Factor (VIF) of each relevant variable ranged from 1.337 to 3.319, all of which were less than 5; the Tolerance values ranged from 0.301 to 0.748, all greater than 0.2. It indicated that there was no serious multicollinearity among the variables.

### Pathway analysis

**Figure 2:**
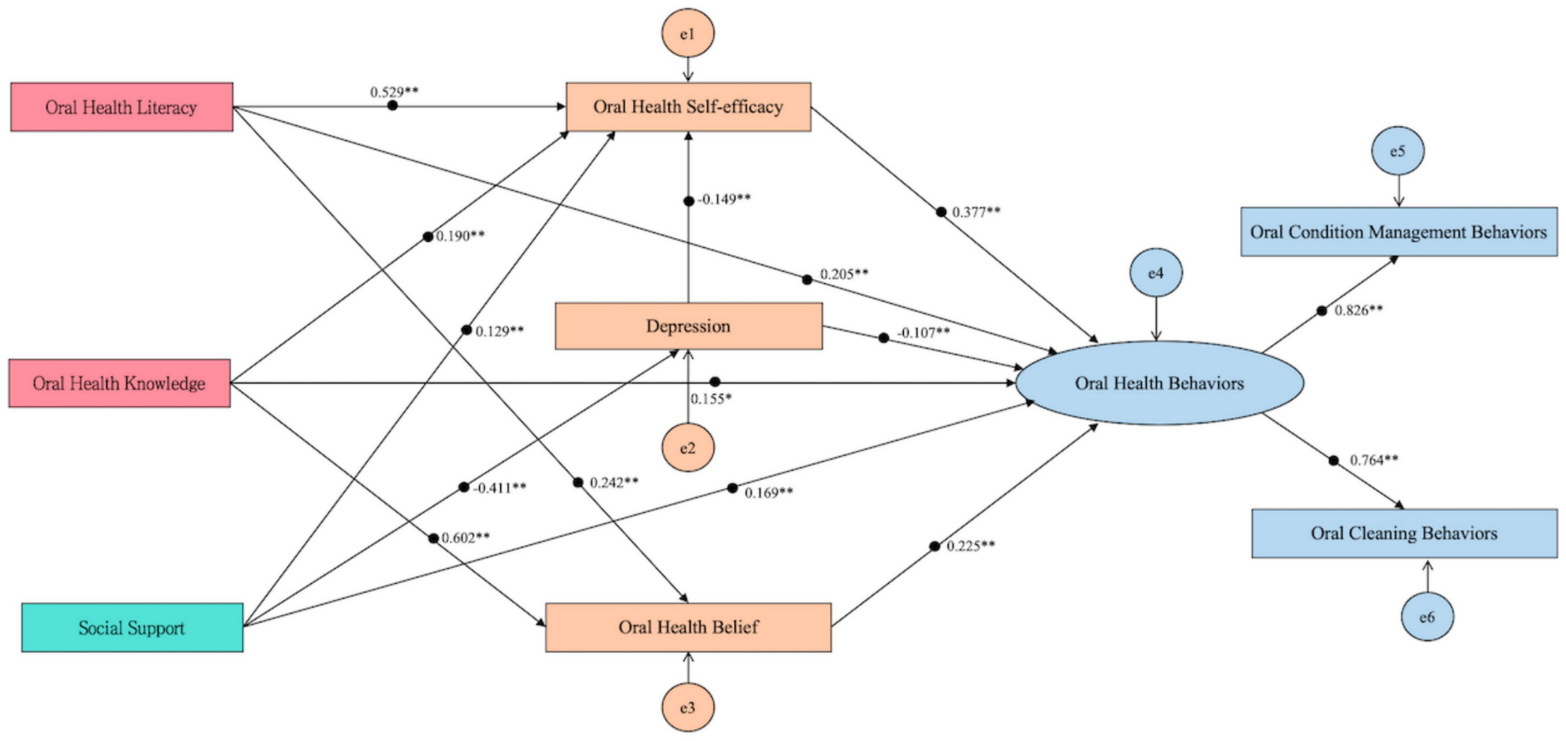
Association pathways of factors related to oral health behaviors in older diabetic patients. *Indicates *p* < 0.05, ** indicates *p* < 0.001, representing the significance level of path coefficients.

Based on the research hypotheses, a structural equation model was constructed. The initially constructed structural equation model showed poor overall fit, with χ^2^/df = 4.174 (on the high side), RMSEA = 0.095 (at the critical threshold), SRMR = 0.023, NFI = 0.977, TLI = 0.955, and CFI = 0.982, indicating the need for model modification. Three statistically non-significant paths (*p* > 0.05), including oral health literacy → depression, oral health knowledge → depression, and social support → oral health belief, were deleted. Based on modification indices (MI) and evidence (24), the correlation path between depression and oral health self-efficacy was added. After modification, the model fit indices were as follows: χ^2^/df = 2.173 (< 3), NFI = 0.987 (> 0.9), TLI = 0.976 (> 0.9), CFI = 0.991 (> 0.9), RMSEA = 0.07 (< 0.08), and SRMR = 0.0474 (< 0.08), suggesting a satisfactory model fit. All paths were statistically significant (*p* < 0.05). Specifically, oral health literacy was positively associated with oral health self-efficacy (*β* = 0.529), oral health beliefs (*β* = 0.242), and oral health behaviors (*β* = 0.205). Oral health knowledge was positively associated with oral health self-efficacy (*β* = 0.190), oral health beliefs (*β* = 0.602), and oral health behaviors (*β* = 0.155). Social support was positively associated with oral health self-efficacy (*β* = 0.129) and oral health behaviors (*β* = 0.169), and was negatively associated with depression (*β* = −0.411). Depression had was negatively associated with oral health self-efficacy (*β* = −0.149) and oral health behaviors (*β* = −0.107). All paths were statistically significant (*p* < 0.05) (see Figure 2). Oral health self-efficacy mediated the relationships among oral health literacy, oral health knowledge, social support, depression, and oral health behaviors. Oral health belief served as correlational links among the relationship between among oral health knowledge, oral health literacy, and oral health behaviors. Depression mediated the relationships between social support and oral health behaviors. The path association results are presented in Table 4.

**Table 4:** Decomposition of path associations of relevant factors on oral health behaviors in older diabetic patients (*N* = 353).

Item	Direct association	Indirect association	Total association
Oral health literacy	0.205	0.254	0.459
Oral health knowledge	0.155	0.207	0.363
Social support	0.169	0.116	0.285
Oral health self-efficacy	0.377	—	0.377
Oral health belief	0.225	—	0.225
Depression	−0.107	−0.056	−0.163

### Common method bias test

Confirmatory factor analysis was employed to test for common method bias. All items were constrained to load onto a single latent factor to construct a one-factor model. The one-factor model yielded a very poor fit (χ^2^/df = 71.928, RMSEA = 0.449, CFI = 0.625, TLI = 0.265, NFI = 0.726), with none of the indices meeting the acceptable criteria. These findings indicated that the variance in the data could not be explained by a single common method factor, suggesting no severe common method bias existed in the present study.

## Discussion

The study analyzed the pathways of factors associated with oral health behaviors among older Chinese patients with diabetes based on the COM-B Model. The results showed that the score of oral health behaviors among older patients with diabetes mellitus was 33.67 ± 9.71 points, which was at a moderate level overall and higher than the national average for the older population (25). The fourth national oral health epidemiology survey found that the overall oral health behaviors of the older in China were at a low level, manifested as insufficient basic oral cleaning and poor medical-seeking behavior (25). The discrepancy may be attributed to the fact that this study was conducted in Shanghai, an economically developed area with superior oral medical resources and more extensive oral health education, enabling local older adults to acquire oral health knowledge and services more conveniently and thus exhibit better oral health behaviors than the national average level (26). The average item score of the oral condition management behaviors dimension was 2.83 ± 0.96 points, which was lower than that of the oral cleaning behaviors dimension 3.47 ± 0.97 points. This indicates that there is considerable room for improvement in patients’ initiative to seek dental treatment. Hyperglycemia can delay wound healing after oral procedures such as tooth extraction and root canal therapy in diabetic patients, increase the risks of infection, bleeding, and other adverse events, and consequently cause patients to fear medical visits (27). However, proactive dental visits contribute to the early prevention and treatment of oral diseases and assist in blood glucose control (28), which is crucial to improving the quality of life in older diabetic patients in their later years (29). Relevant factor analysis serves as the fundamental premise for developing the intervention measures. The pathways analysis of factors associated with oral health behaviors among older diabetic patients can provide evidence for the formulation of targeted intervention strategies.

Univariate analysis showed that among demographic factors, older diabetic patients with younger age, female gender, married status, higher educational level, better economic status, mental labor occupation, and urban resident medical insurance obtained higher scores of oral health behaviors. The differences above may be explained as follows: younger and more educated patients have more capabilities and channels to acquire oral health knowledge and show higher initiative in maintaining oral health (30); compared with male patients, female individuals pay more attention to physical health and external appearance, and present stronger self-management awareness and behavioral initiative for oral health (31); married older patients can obtain sufficient spousal support and receive better supervision on daily oral health behaviors than unmarried ones (32); patients with better economic conditions have stronger financial capacity for regular oral health maintenance (33); compared with manual workers, mental workers generally have higher educational attainment and better economic conditions, which increases their likelihood of adopting oral health behaviors (30, 33); patients covered by urban resident medical insurance enjoy a higher reimbursement ratio for dental visits, thereby reducing their economic burden (34). In terms of disease-related factors, patients with shorter disease duration, fewer complications, better blood glucose control and fewer chronic comorbidities presented a higher level of oral health behaviors. This may be attributed to less physical discomfort, lower complexity of disease treatment, and reduced psychological pressure in such patients, allowing them to devote more effort to oral cleaning, regular oral examinations, and other health-related behaviors (35).

This study found that capability factors (oral health literacy and oral health knowledge), opportunity factor (social support), and motivational factors (oral health self-efficacy, oral health belief, and depression) were associated with oral health behaviors, which verified Hypothesis 1. Oral health literacy showed a positive association with oral health behaviors (*β* = 0.205), which aligns with previous research results (6). Oral health literacy helps patients to master standardized oral cleaning methods, knowledge on the prevention and control of diabetes-related oral diseases, and scientific timing of medical visits (36). Patients with high literacy levels not only pay more attention to maintaining their own oral health but also gain a deeper understanding of the significance of oral health for blood glucose management, which is linked to higher adherence in oral health behaviors (36). Oral health knowledge was positively associated with oral health behaviors (*β* = 0.155). Sufficient knowledge reserve helps patients to accurately identify common oral diseases in older diabetic individuals, understand the correlation between blood glucose control and oral conditions, and master scientific oral cleaning methods, which not only deepens their perception of the necessity of such behaviors but also reduces misconceptions in practice, thereby providing an important foundation for the initiation and maintenance of health behaviors (37). This result is consistent with previous research findings among populations such as community-dwelling older (38) and stroke patients (39). Social support was positively correlated with oral health behaviors (*β* = 0.169). Patients’ family members, friends, and other relatives can encourage patients to adopt positive oral health behaviors by setting an example (40). Meanwhile, financial support from family members can help reduce the economic burden of dental treatment for older retirees. Oral health self-efficacy was positively associated with oral health behaviors (β = 0.377), which is consistent with previous research results (10), and oral health self-efficacy showed the closest association with oral health behaviors and was closely related to the arrangement of oral health behaviors. Oral health behaviors are dynamic decision-making process, and high oral health self-efficacy can significantly enhance patients’ awareness and practical ability of self-health management (10). Older diabetic patients are at a higher risk of oral diseases, and blood glucose fluctuations tend to aggravate oral discomfort. Individuals with high self-efficacy tend to have better adherence to standardized oral hygiene routines in daily life and show stronger willingness and a more proactive attitude to seek medical care when oral abnormalities occur. Oral health belief was positively associated with oral health behaviors (*β* = 0.225). Health belief is directly related to the initiation, persistence, and standardization of health behaviors (41). Older diabetic patients are mostly characterized by a long course of disease, comorbidity of multiple illnesses, and cognitive decline (2). These factors may relate to patients’ oral health belief and become potential barriers to their oral health behaviors. Depression was negatively associated with the older diabetic patients’ oral health behaviors (*β* = −0.107). Depressed older diabetic patients exhibit reduced motivation, attention, and persistence in maintaining oral health (9). They are more likely to hold a negative attitude toward life and tend to overlook the importance of oral health for blood glucose control and physical well-being, with relatively lower levels of oral health behaviors (9, 42).

Among motivational factors, oral health self-efficacy (*β* = 0.199 and 0.072) and oral health belief (*β* = 0.054 and 0.135) both formed associative pathways connecting on capability factors (oral health literacy and oral health knowledge) and oral health behaviors, which verified Hypothesis 2. Oral health knowledge and oral health literacy provide a cognitive basis for self-efficacy. A higher level of cognition about the relationship between oral health and diabetes, as well as relevant oral health maintenance needs, is positively associated with stronger comprehensive capabilities in oral cleaning, disease judgment, and medical consultation. Accordingly, they possess a greater sense of control and confidence in daily oral care and oral disease management, accompanied by higher self-efficacy and an increased likelihood of engaging in positive oral health behaviors (10, 43). In addition, oral health literacy and knowledge can help patients recognize that, compared with the general older population, diabetes significantly increases their susceptibility to oral diseases and the severity of the condition, reduce the perceived barriers to unreasonable oral disease management behaviors, form positive oral health belief, and thus prompt patients to adopt oral health behaviors in daily life (44, 45). The study also found that the overall association of oral health literacy on oral health behaviors was the highest among all factors (*β* = 0.459), indicating that it was the most important factor linked to oral health behaviors and an important entry point for conducting oral health interventions.

Among motivational factors, oral health self-efficacy exhibited the correlational linkage between opportunity factor (social support) and oral health behaviors (*β* = 0.049); depression also presented a correlational linkage between social support and oral health behaviors (*β* = −0.044), which verified Hypothesis 3. As a high-risk group for oral diseases, older diabetic patients with a high level of social support receive more practical material support, information support, and emotional support from the outside world, which helps them obtain more social resources. When facing oral diseases, these patients have higher confidence in seeking medical treatment and performing daily oral hygiene, along with a higher level of health behaviors (40). This indirect association has not been reported in previous studies, and this study enriches the relational pathway between social support, oral health self-efficacy, and oral health behaviors. Older diabetic patients are a high-stress group who suffer from long-term chronic disease, oral discomfort, and declining self-care ability. Favorable social support is linked to lower disease-related psychological stress, decreased depression risk, and greater attention to oral health, a higher likelihood of adopting oral health behaviors (46). In addition, among motivational factors, depression was indirectly associated with the patients’ oral health behaviors through oral health self-efficacy (*β* = −0.056). Depressive symptoms are correlated with low mood and diminished attention to personal health, along with negative perceptions such as helplessness and low behavioral necessity, which are linked to lower confidence in oral health maintenance, reduced oral health self-efficacy, and poorer oral health behaviors (47).

### Research implications and suggestions for future research

This study provides empirical support for the utilization of the COM-B model within the field of oral health behaviors in older diabetic patients and validates the three research hypotheses proposed herein, offering a scientific basis for the oral health management of this population. In future practice, medical staff should formulate individualized intervention strategies for oral health behaviors according to the demographic characteristics of older patients with diabetes, including age, educational level, economic status, duration of diabetes, diabetic complications, and comorbid chronic diseases, et al. Relying on community health services and chronic disease clinics, regular oral health education should be carried out for older patients with diabetes to popularize knowledge about oral hygiene and the prevention and treatment of diabetes-related oral diseases, and to train patients in oral cleaning skills so as to improve their comprehensive health management ability. Family members should be encouraged to provide supervision and companionship. Communities can add basic dental services, such as oral disease screening, to reduce patients’ medical costs and travel burdens. Targeted psychological counseling shall be delivered to relieve negative emotions. Combined with health science popularization and positive cases, patients can fully understand the correlation between oral health and blood glucose control, thereby optimizing their health cognition. Practical operation demonstration can be adopted to enhance patients’ oral health self-efficacy.

Future longitudinal studies with multi-center and large-sample designs are warranted to further clarify the associations between oral health knowledge, oral health literacy, social support, oral health self-efficacy, oral health belief, depression, and oral health behaviors in older diabetic patients. In addition, subgroup analysis can be carried out for populations with different demographic and disease characteristics to identify the specificity of oral health behaviors in different subgroups and optimize the intervention priorities for different populations.

### Strengths and limitations

Guided by the COM-B model, this study analyzed the direct and indirect association pathways of factors related to oral health behaviors among older diabetic patients from a multi-dimensional perspective and provided a basis for constructing oral health intervention strategies and improving the oral health management systems for this population. Meanwhile, the participants were recruited from both hospitals and communities, which renders certain reference value for public health practice. However, our study employed a cross-sectional design. The patients were recruited from one tertiary hospital and a community health service center, and a stratified sampling method was not adopted, resulting in certain constraints on sample representativeness. Meanwhile, it is impossible to clarify the causal relationship between various factors and oral health behaviors, which requires further research in the future. In addition, demographic and disease-related indicators were only included for preliminary screening in the univariate analysis of this study. The above confounding variables were not further incorporated into the structural equation model for multivariate correction analysis. Future studies could further optimize the research model by incorporating demographic and disease characteristic variables for confounding adjustment.

## Conclusion

This study demonstrated that the older diabetic patients’ oral health behaviors are at a moderate level, with considerable room for improvement. Capability factors (oral health literacy, oral health knowledge), opportunity factor (social support) and motivational factors (oral health belief, oral health self-efficacy, and depression) are directly or indirectly associated with oral health behaviors. For clinical practice, this relevant factor model can be used as a basis to formulate targeted intervention strategies, helping older diabetic patients improve oral health behaviors and reduce adverse oral health-related outcomes. The study is a cross-sectional design involving patients recruited from one tertiary hospital and one community health service center. The sample representation has certain limitations, and the cause-effect link between each factor and oral health behaviors cannot be clarified. Further research is needed in the long term.

## Data Availability

The raw data supporting the conclusions of this article will be made available by the authors, without undue reservation.
